# Impaired IL‐35/Treg Axis Facilitates Neuropathic Pain Via TNF‐α, TLR4, and HMGB1 Activation

**DOI:** 10.1002/iid3.70416

**Published:** 2026-05-24

**Authors:** Pingdi You, Lirui Shen, Zhixiong Huang, Cuihua Yuan, Cuihua Wei, Sunzhi Lin, Bili Wang, Yongwu Chen

**Affiliations:** ^1^ Department of Pain Medicine Mindong Hospital Affiliated to Fujian Medical University Fu'an China; ^2^ Department of Pathology Mindong Hospital Affiliated to Fujian Medical University Fu'an China; ^3^ Department of Orthopedics Mindong Hospital Affiliated to Fujian Medical University Fu'an China

**Keywords:** inflammatory reaction, interleukin 35, neuropathic pain, Tregs immunity

## Abstract

**Background:**

Neuropathic pain (NP) is a chronic condition characterized by persistent pain due to dysfunction in the peripheral or central nervous system. Recent studies suggest immune dysregulation, particularly involving regulatory T cells (Tregs) and inflammatory mediators, plays a significant role in NP. Interleukin‐35 (IL‐35), an anti‐inflammatory cytokine secreted by Tregs, has shown potential immunosuppressive effects, but its role in NP remains unclear. This study aimed to investigate the expression and regulatory role of IL‐35 on Treg‐mediated immune modulation and inflammatory responses in a rat model of neuropathic pain.

**Methods:**

A total of 18 male Sprague–Dawley rats were randomly assigned to Control, Sham, and Neuropathic Pain (NP) groups. The NP model was established using chronic constriction injury (CCI) of the sciatic nerve. Pain behaviors were assessed by measuring mechanical withdrawal threshold (MWT) and thermal withdrawal latency (TWL) at multiple time points post‐surgery. IL‐35 levels in serum were detected via ELISA. Flow cytometry was used to evaluate CD4⁺ and CD8⁺ T cell populations. Histopathological analysis of spinal cord tissue was performed using HE staining. Expression of IL‐35, TNF‐α, TLR4, and HMGB1 in spinal cord tissues was evaluated by immunohistochemistry, Western blotting, and RT‐PCR.

**Results:**

Rats in the NP group exhibited significantly reduced MWT and TWL, confirming successful model establishment. Serum IL‐35 levels and CD4⁺ T cell counts were significantly decreased, whereas CD8⁺ T cell counts were increased compared to controls (*p* < 0.05). Histopathology revealed neuronal degeneration in the NP group's spinal dorsal horn. In spinal cord tissue, IL‐35 protein and mRNA levels were significantly downregulated, while TNF‐α, TLR4, and HMGB1 were upregulated at both protein and mRNA levels (*p* < 0.05).

**Conclusion:**

IL‐35 expression is diminished in neuropathic pain and correlates with reduced Treg‐mediated immunosuppressive function and increased neuroinflammation. The data suggest IL‐35 mitigates neuropathic pain through modulation of T cell responses and suppression of TNF‐α, TLR4, and HMGB1 signaling pathways. IL‐35 may serve as a potential therapeutic target for neuropathic pain management.

## 1 Introduction

Neuropathic pain (NP) is a common and debilitating chronic pain condition caused by direct damage or dysfunction of the peripheral or central nervous system. It is clinically hallmarked by symptoms of spontaneous pain, hyperalgesia (sensitivity to painful stimuli), and allodynia (pain to stimuli that are normally painless) [1, 2]. In addition to its sensory symptoms, NP is frequently associated with serious psychological comorbidities, such as anxiety, depression, and sleep disturbances, which collectively significantly compromise the patient's quality of life. The chronic and refractory nature of NP has prompted its acknowledgment as a serious worldwide health burden, both economically and socially. Despite the progress in pain research, the exact pathophysiological mechanisms of NP are still not fully elucidated. Gathering evidence indicates that NP is not a nervous system disorder alone but a multifaceted neuroimmune disorder with complex interactions among neuronal cells, glial cells (astrocytes and microglia), immune cells, cytokines, and chemokines in the peripheral and central nervous systems. Glial cell activation in the spinal dorsal horn following nerve injury is critically involved in the amplification and maintenance of pain through the release of pro‐inflammatory mediators such as tumor necrosis factor‐alpha (TNF‐α), interleukin‐1 beta (IL‐1β), and other cytokines [3, 4]. This neuroinflammatory response induces neuronal hyperexcitability and central sensitization, major factors for the chronicity of neuropathic pain. Among the numerous immune regulatory elements involved in NP, regulatory T cells (Tregs) have attracted growing interest because they play a fundamental role in preserving immune homeostasis and limiting excessive inflammation. Tregs perform their immunosuppressive actions by secreting anti‐inflammatory cytokines like interleukin‐10 (IL‐10), transforming growth factor‐beta (TGF‐β), and more recently discovered interleukin‐35 (IL‐35). Research has demonstrated that augmenting Treg numbers can reduce neuropathic pain symptoms in experimental models, pointing to their potential therapeutic significance [5]. Interleukin‐35 (IL‐35) is a heterodimeric cytokine made up of Epstein–Barr virus‐induced gene 3 (EBI3) and IL‐12p35 subunits. It is mainly secreted by Tregs and is critical for their optimal immunosuppressive function. IL‐35 has been shown to play a role in the modulation of immune responses in several pathological conditions, such as autoimmune diseases, infectious diseases, and cancer. While IL‐35 expression is generally low or undetectable in peripheral blood Tregs and other T cell populations under physiological conditions [6, 7], it may be increased upon immune challenge. In response to stimulation, IL‐35 promotes the expansion of a novel subset of inducible regulatory T cells called iTr35. These cells further amplify an anti‐inflammatory environment by producing IL‐35 in addition to IL‐10 and TGF‐β, thus suppressing effector T cell proliferation and cytokine secretion potently [8]. Through these processes, IL‐35 helps dampen excessive immune responses and promote immune tolerance. Still, the definite role and mechanistic pathways of IL‐35 in neuropathic pain are not well understood. Although its anti‐inflammatory function predicts a protective effect, how it interacts with glial cell‐mediated neuroinflammation and T cell immune regulation in NP is not well characterized. In addition, High mobility group box 1 (HMGB1) acts as a damage‐associated molecular pattern (DAMP) that is released extracellularly following nerve injury. Extracellular HMGB1 binds to Toll‐like receptor 4 (TLR4), activating downstream inflammatory signaling pathways that promote the production of pro‐inflammatory cytokines, including tumor necrosis factor‐α (TNF‐α). This HMGB1–TLR4–TNF‐α axis plays a critical role in sustaining neuroinflammation and neuropathic pain. Thus, the present study is intended to systematically explore the expression profile of IL‐35 in a well‐characterized rat model of neuropathic pain and reveal its regulatory role in T cell‐mediated immune responses and major inflammatory pathways.

## 2 Methods

### 2.1 Experimental Animals

Eighteen male SD rats, aged 6–7 weeks, were purchased from Spefford (Beijing Biotechnology Co. Ltd.) with a license number SCXK (Beijing) 2019‐0010. The rats were all SPF grade and weighed 200–220 g. The rats were housed in a standardized animal room maintained at a consistent temperature of 22°C–25°C and a relative humidity of 50%–60%. The indoor lighting alternated every 12 h between day and night settings, ensuring free access to both water and food. They were fed adaptively for 1 week.

### 2.2 Reagents and Instruments

Rabbit/mouse IgG‐two‐step immunohistochemistry kit, DAB chromogenic kit (Product No.: 18B13B04, 17J20B27, Wuhan Boster Biological Co. Ltd.); HE staining kit (Product No.: C0105S, Shanghai Biyuntian Biological Co. Ltd.); TNF‐α antibody (Cat. No.: AF1015, Affinity Company); TLR4 antibody, HMGB1 antibody, GAPDH antibody (Cat. No.: 33650‐1‐Ig, 66525‐1‐Ig, 60004‐1‐Ig, Proteintech Company); IL‐35 Antibody (Cat. No.: ab133751, Abcam, USA); CD3‐FITC, CD8‐perCP (Cat. No.: 201403, 201712, Biolegend); CD4‐APC (Cat. No.: AR00450, Lianke Biotech); Flow cytometer (Model: FACSCalbur, BD Company); desktop low‐speed centrifuge (model: L500‐A, Hunan Xiangyi Laboratory Instrument Development Co. Ltd.); slice baking machine, automatic tissue dehydration machine (model: KH‐P2, KH‐TS, Xiaogan, Hubei) Kuohai Medical Technology Co. Ltd.); Rat plantar allodynia meter (Model: YLS‐22A, Jinan Yiyan Technology Development Co. Ltd.); Biological microscope (Model: E200MV, NIiKon Company); PCR instrument (Model: PTC100, United States BIO‐RAD Corporation).

### 2.3 Grouping and Model Preparation

Eighteen rats were randomly divided into a normal control group (Control group), a sham operation group (Sham group) and a neuropathic pain rat model group (NP group), with six rats in each group. The model for chronic compression injury (CCI) of the sciatic nerve was established in alignment with the methodology delineated in reference [9] to simulate clinical scenarios of neuropathic pain. Anesthetize by intraperitoneal injection of 10% chloral hydrate 35 mg/kg, fix the rat on the operating table, remove the hair on the right hind limb of the rat, disinfect the skin regularly, incise the skin along the femur, bluntly separate the muscles, fully expose the sciatic nerve, and use 4–0 absorbable surgical suture to tie 4 loose knots before the sciatic nerve bifurcation, with an interval of 1 mm so that the epineurium is slightly compressed. The tightness is suitable for a slight twitching of the thigh muscles or toes and does not affect the blood supply of the epineurium, not too tight; then suture the muscles and skin layer by layer, clean the incision and cover it with penicillin to prevent infection. In the sham group, the sciatic nerve was exposed for a duration of 2–3 min without implementing any constriction, and the rest of the operations were the same as in the NP group. The control group was normal rats without any operation.

### 2.4 Observation and Measurement of Pain Behavior

Prior to model establishment, the baseline pain threshold for rats in each group was assessed. Rats were placed in the testing apparatus in advance to allow acclimation, reduce stress‐related behavioral responses, and ensure stable baseline pain measurements. Subsequent to modeling, pain behavior was evaluated at intervals on days 1, 3, 5, 7, and 14, specifically between 8:00 and 12:00 a.m. The mechanical withdrawal threshold (MWT) for rats in each group was quantified utilizing an electronic algometer. MWT testing began with a von Frey filament exerting a force of 2 g. If no withdrawal response was observed, progressively higher forces were applied; if a positive response occurred, a lower force was subsequently tested. This up‐down method was continued until the mechanical withdrawal threshold was determined. During the measurement, the rats needed to be placed in the measurement box 15 min in advance. In order to ensure that the rats were in a relaxed state during the measurement, after the rats had no grooming and exploratory behavior, von Frey filaments were used to vertically stimulate the middle part of the plantar of the rats for a duration of ≤ 6 s. A positive response was identified upon the exhibition of foot lifting or licking behavior by the rats. The measurement first starts from 2 g. If a stimulus of a given intensity fails to elicit a positive response, the next higher intensity is applied; conversely, if a positive response is observed, the preceding lower intensity is administered. This continues until the first positive reaction occurs. reaction, and then measure 4 times continuously, with an interval of 30 s each time.

Thermal withdrawal latency (TWL) was assessed using a radiant heat pain meter (YLS‐22A, Jinan Yiyan Technology Development Co. Ltd., China). Rats were placed in the transparent plexiglass box 30 min in advance for acclimation, and behavioral testing was initiated after an additional 15‐min adaptation period. The radiant heat intensity was adjusted to produce a baseline latency of approximately 15 s, with a cutoff time of 18 s to prevent tissue damage. A thermal pain meter is set up and the radiant heat intensity is maintained to ensure that the rat's basic value is maintained at 15 s, and the thermal radiation exposure time is 18 s. When the rat shrinks its foot, turn off the radiant heat light source. Otherwise, continue to irradiate it for 18 s. The paw withdrawal reflex latency of rats in each group was recorded, and the measurement was repeated 3 times with an interval of 5 min each time. During the 5‐min intervals between TWL measurements, the paw was allowed to cool naturally and return to baseline conditions. No visible tissue damage or burns were observed. Mechanical and thermal pain behaviors were assessed on the same day, with sufficient rest intervals to minimize potential interference.

### 2.5 Specimen Collection and Measurement

Following the pain behavior assessment, which concluded on the 15th day post‐modeling, 3 mL of blood was extracted from the aorta of rats across all groups. One part was stored in a −20°C refrigerator, and the other part was placed in a non‐anticoagulation tube and left to stand in a normal temperature environment for 1 h, centrifuged at a speed of 1500 r/min for 30 min, and serum was collected. IL‐35 levels in serum were detected via ELISA. Subsequent to this, rats were anesthetized and euthanized. L4‐5 spinal cord tissue from each group was extracted, with one portion being preserved in a 4% paraformaldehyde solution and the other immediately placed in liquid nitrogen, before being transferred to storage at −80°C.

### 2.6 Flow Cytometric Analysis of CD4⁺ and CD8⁺ T Lymphocyte Populations

Take 100 μL of whole blood from rats in each group and place it in a 2 mL heparin‐free EP tube. Add 2 μL of CD4+ antibody and 5 μL of CD3+ and CD8+ antibodies, respectively. Shake thoroughly and evenly, and react at room temperature in the dark for 15 min. Add 1.5 mL of red blood cell lysate to the sample for staining. After mixing, incubate in the dark at 4°C for 15 min. Centrifuge at 1000 r/min for 1 min. Collect and discard the supernatant. Wash the precipitate with PBS solution. Once, 0.5 mL of PBS was diluted and precipitated, and the CD4+ and CD8+ levels in the whole blood of rats in each group were detected on a flow cytometer.

### 2.7 Hematoxylin–Eosin (HE) Staining for Spinal Cord Histopathological Assessment

Take out the spinal cord tissue soaked in 4% paraformaldehyde solution, embed the sample in paraffin, cut it into 4 μm thick slices with a microtome, dehydrate the tissue slices with alcohol, place the tissue slices in xylene to make them transparent, add hematoxylin to the tissue slices, rinse with distilled water and then dehydrate with alcohol. Stain the sections with eosin for 3 min and observe them under a microscope.

### 2.8 Immunohistochemical Analysis of IL‐35, TNF‐α, TLR4, and HMGB1 Expression in Spinal Cord Tissue

Dewax the spinal cord tissue sections to water, then perform endogenous inactivation, antigen retrieval and blocking on the paraffin sections, and then add primary antibodies IL‐35, TNF‐α, TLR4, and HMGB1 (1:1000), and incubate at 4°C. Incubate overnight; then add secondary antibody (1:1000) for incubation, wash with PBS, use DAB for color development, and then perform hematoxylin counterstaining, dehydrate and clear the sample, and observe under a microscope after sealing. In spinal cord tissue, positive cells showed tan granules in the cytoplasm.

### 2.9 Western Blot Analysis of IL‐35, TNF‐α, TLR4, and HMGB1 Protein Expression in Spinal Cord Tissue

The BCA method was used to determine the total protein concentration in the spinal cord tissue of rats in each group. Boil the protein sample with a final concentration of 2 μg/mL in hot water and store it in a −20°C refrigerator for 10 min. After thoroughly mixing 50 μg protein sample with the loading buffer, boil it in boiling water to denature it. Protein samples underwent separation via SDS‐PAGE and were transferred to a PVDF membrane, which was then blocked with 5% skim milk for 1 h. Subsequently, the membrane was washed with Tris‐buffered saline solution (TBST) inclusive of 1 mL/L Tween and assayed for IL‐35. TNF‐α, TLR4, and HMGB1 (at a 1:2000 dilution) were incubated at 4°C overnight, followed by a thorough washing of the PVDF membrane with a detergent solution. The membrane was treated with a secondary antibody at a dilution of 1:5000 and incubated at room temperature for 2 h. Subsequently, ECL luminescent solution was applied to facilitate development and exposure. The band intensities were then analyzed for grayscale values utilizing Image Lab software.

### 2.10 Detection of IL‐35, TNF‐α, TLR4, and HMGB1 mRNA Expression in Spinal Cord Tissue by RT‐PCR

RNA was extracted from the spinal cord tissue following the guidelines provided by the RNAzol RT kit, and subsequent measurement of the RNA concentration was conducted. Per the instructions of the reverse transcription kit, a reverse transcription reaction was conducted on the extracted total RNA to synthesize the corresponding cDNA. Instantly centrifuge each primer (see Table 1 for primer sequences), add 6.8 μL of Mix solution, 0.4 μL of each upstream and downstream primer, and 2.0 μL of cDNA template to the primer, and finally add deionized water to 10 μL. Primer amplification: the primers were pre‐denatured at 95°C for 1 min; denatured at 95°C for 20 s; annealed at 56°C for 20 s; extended at 72°C for 30 s; a total of 40 cycles. The 2‐CT method was used to examine the levels of target gene expression.

**TABLE 1:** Primer sequence list.

Gene	Sequence list
IL‐35	F:5′‐CCCTGTGCCTTGGTAG‐3′
	R:5′‐AGAGTCTCGCCGCTGT‐3′
TNF‐α	F:5′‐CTCAAGCCCTGGTATGAGCC‐3′
	R:5′‐CTCCAAAGTAGACCTGCCCG‐3′
TLR4	F:5′‐GGCAGCAGGTCGAATTGTAT‐3′
	R:5′‐GCTTCTTGTTCTTCCTCTGATGT‐3′
HMGB1	F:5′‐TGTGCAAACTTGTCGGGAGGA‐3′
	R:5′‐TCTTTCATAACGGGCCTTGTCC‐3′
GAPDH	F:5′‐ACAGCAACAGGGTGGTGGAC‐3′
	R:5′‐TTTGAGGGTGCAGCGAACTT‐3′

### 2.11 Statistical Analysis

Experimental data were subject to statistical analysis utilizing GraphPad Prism 8.0 software, with measurement data being expressed as mean ± standard deviation ($\bar{X}$ ± s). Inter‐group data were statistically compared using one‐way ANOVA, while pairwise differences between groups were assessed through the LSD‐t test. Based on the experimental outcomes, a *p*‐value less than 0.05 denotes a statistically significant difference. All behavioral and molecular experiments were performed using six rats per group, and Western blot analyses were repeated at least 3 times independently.

## 3 Results

### 3.1 Mechanical and Thermal Pain Thresholds in Each Group

**FIGURE 1:**
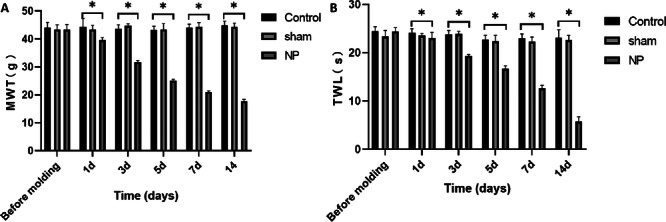
Comparison of mechanical pain threshold and thermal pain threshold of rats in each group. (A) Comparison of mechanical pain thresholds of rats in various groups at different times. (B) Comparison of thermal pain thresholds of rats in various groups at different times. *p < 0.05.

Prior to model establishment, there were no statistically significant differences in MWT and TWL among the Control, Sham, and NP groups (*p* > 0.05). Following the surgical procedure, both the Control and Sham groups maintained stable MWT and TWL across the observation period (*p* > 0.05). In contrast, rats in the NP group exhibited a progressive and statistically significant reduction in both mechanical and thermal thresholds post‐surgery, indicative of successful neuropathic pain modeling. Compared to the Control group, the NP group showed markedly lower MWT and TWL values on days 1, 3, 5, 7, and 14 (*p* < 0.05), confirming the presence of mechanical hypersensitivity and thermal hyperalgesia characteristic of neuropathic pain (Figure 1).

### 3.2 Serum IL‐35 Levels in Each Group

**FIGURE 2:**
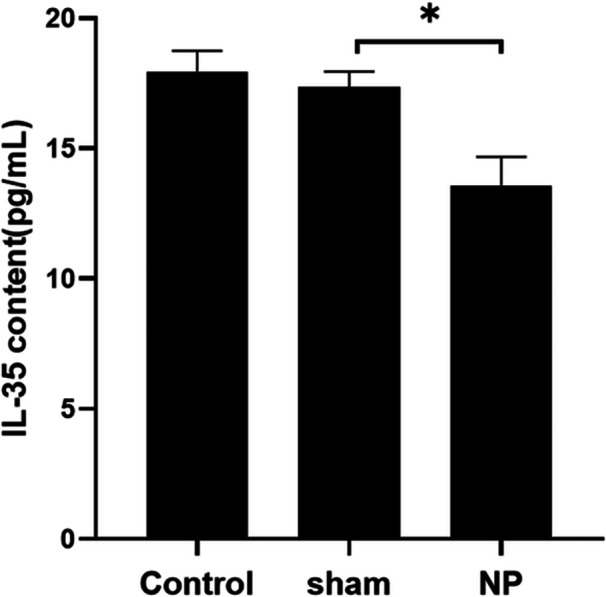
Comparison of IL‐35 levels in serum of rats in each group. *p < 0.05.

Serum analysis revealed no significant difference in IL‐35 levels between the Control and Sham groups (*p* > 0.05). However, rats in the NP group exhibited a statistically significant reduction in circulating IL‐35 levels compared to the Control group (*p* < 0.05). This reduction suggests that neuropathic pain is associated with a suppression of systemic anti‐inflammatory mediators such as IL‐35 (Figure 2).

### 3.3 CD4⁺ and CD8⁺ T Cell Populations in Peripheral Blood

**FIGURE 3:**
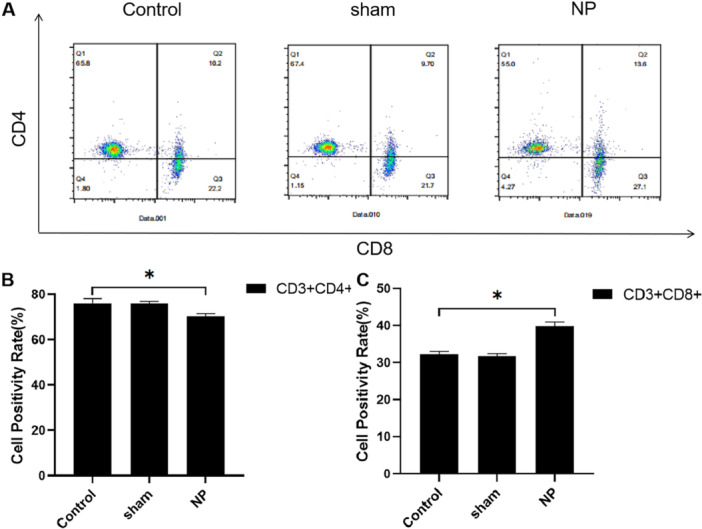
Comparison of CD4+ and CD8+ levels in whole blood of rats in each group. (A) The CD4+ and CD8+ levels in rats detected by Flow cytometry. (B) Comparison of CD4+ level in whole blood of rats in each group. (C) Comparison of CD8+ level in whole blood of rats in each group. *p < 0.05.

Flow cytometry analysis showed no significant differences in CD4⁺ and CD8⁺ T lymphocyte levels between the Control and Sham groups (*p* > 0.05). However, the NP group demonstrated a significant decrease in CD4⁺ T cell levels and a concomitant increase in CD8⁺ T cell levels when compared to the Control group (*p* < 0.05). These findings indicate a shift toward a pro‐inflammatory immune profile in neuropathic pain conditions, characterized by reduced regulatory T cell populations and elevated cytotoxic T cell responses (Figure 3).

### 3.4 Histopathological Changes in Spinal Cord Tissue

**FIGURE 4:**
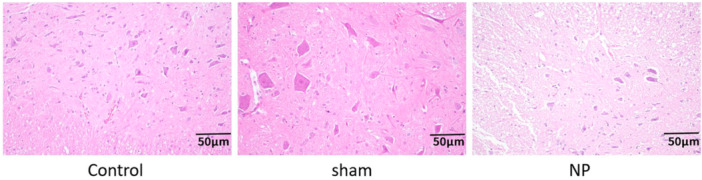
Histopathological changes in rat spinal cord (×200) by HE staining.

Histological examination of spinal cord sections revealed well‐preserved neuronal morphology and dense cellular organization in both the Control and Sham groups. In contrast, spinal cord sections from the NP group exhibited marked pathological alterations, including neuronal shrinkage, nuclear pyknosis, cellular degeneration, and evidence of tissue loosening, all of which are consistent with neuronal injury and neuroinflammation associated with chronic neuropathic pain (Figure 4).

### 3.5 Immunohistochemical Expression of IL‐35, TNF‐α, TLR4, and HMGB1

**FIGURE 5:**
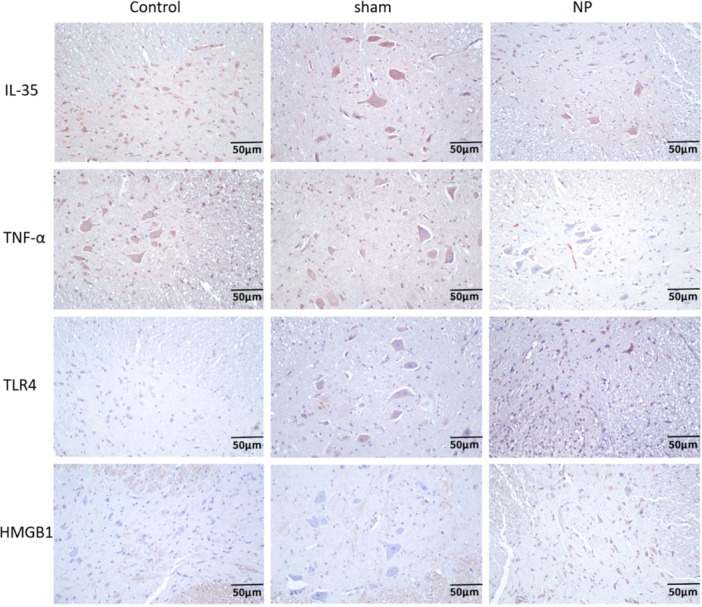
The positive expression of IL‐35, TNF‐α, TLR4, and HMGB1 in spinal cord tissue by immunohistochemistry detecting (×200).

Immunohistochemistry analysis revealed no significant differences in the expression of IL‐35, TNF‐α, TLR4, and HMGB1 between the Control and Sham groups (*p* > 0.05). In the NP group, however, there was a notable decrease in IL‐35‐positive staining within spinal cord tissues compared to controls. Conversely, there was a significant increase in the positive expression of pro‐inflammatory mediators TNF‐α, TLR4, and HMGB1 (*p* < 0.05), suggesting activation of inflammatory signaling pathways in neuropathic pain (Figure 5).

### 3.6 Protein Expression of IL‐35, TNF‐α, TLR4, and HMGB1 in Spinal Cord Tissue

**FIGURE 6:**
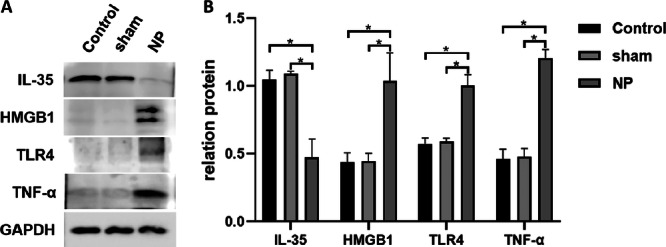
Comparison of IL‐35, TNF‐α, TLR4, and HMGB1 protein expression in spinal cord tissue of rats in each group. (A) IL‐35, TNF‐α, TLR4, and HMGB1 protein expression in spinal cord tissue. (B) Comparison of IL‐35, TNF‐α, TLR4, and HMGB1 protein expression in spinal cord tissue of rats in each group. *p < 0.05.

Western blot analysis confirmed the immunohistochemical findings. Rats in the NP group showed significantly reduced IL‐35 protein expression in the spinal cord compared to the Control group (*p* < 0.05). Conversely, the NP group exhibited significantly elevated protein levels of TNF‐α, TLR4, and HMGB1, further corroborating the activation of inflammatory signaling cascades in neuropathic conditions. Protein expression levels in the Sham group were comparable to those in the Control group (*p* > 0.05). The presence of two HMGB1 bands likely reflects different molecular or redox forms of HMGB1, which have been shown to exhibit distinct biological activities (Figure 6).

### 3.7 mRNA Expression of IL‐35, TNF‐α, TLR4, and HMGB1 in Spinal Cord Tissue

**FIGURE 7:**
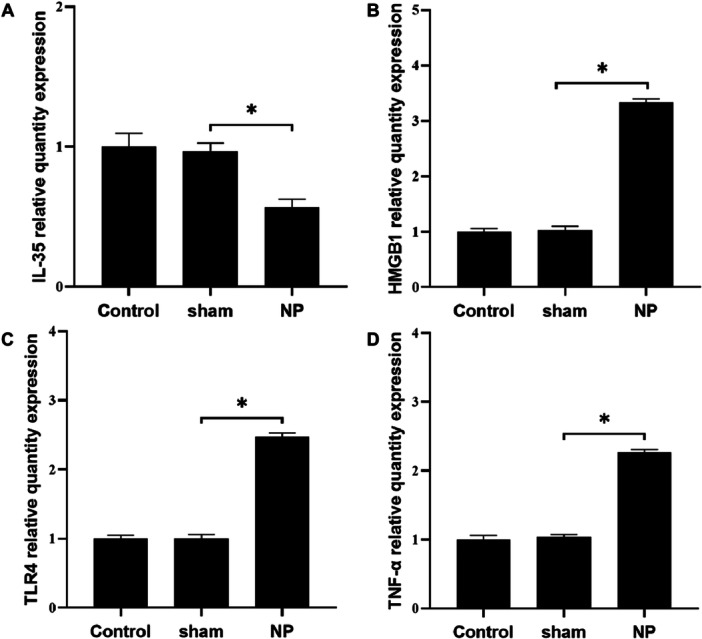
Comparison of IL‐35, TNF‐α, TLR4, and HMGB1 mRNA expression in spinal cord tissue of rats in each group. (A) Comparison of IL‐35 mRNA expression in spinal cord tissue. (B) Comparison of HMGB1 mRNA expression in spinal cord tissue. (C) Comparison of TLR4 mRNA expression in spinal cord tissue. (D) Comparison of TNF‐α mRNA expression in spinal cord tissue. *p < 0.05.

RT‐PCR analysis demonstrated similar trends at the transcriptional level. The NP group exhibited significantly lower IL‐35 mRNA expression relative to the Control group (*p* < 0.05). In contrast, mRNA expression levels of TNF‐α, TLR4, and HMGB1 were significantly upregulated in the NP group compared to the Control group (*p* < 0.05). There were no statistically significant differences in gene expression between the Control and Sham groups (*p* > 0.05), indicating that the observed changes were specific to neuropathic injury rather than surgical manipulation alone (Figure 7).

**FIGURE 8:**
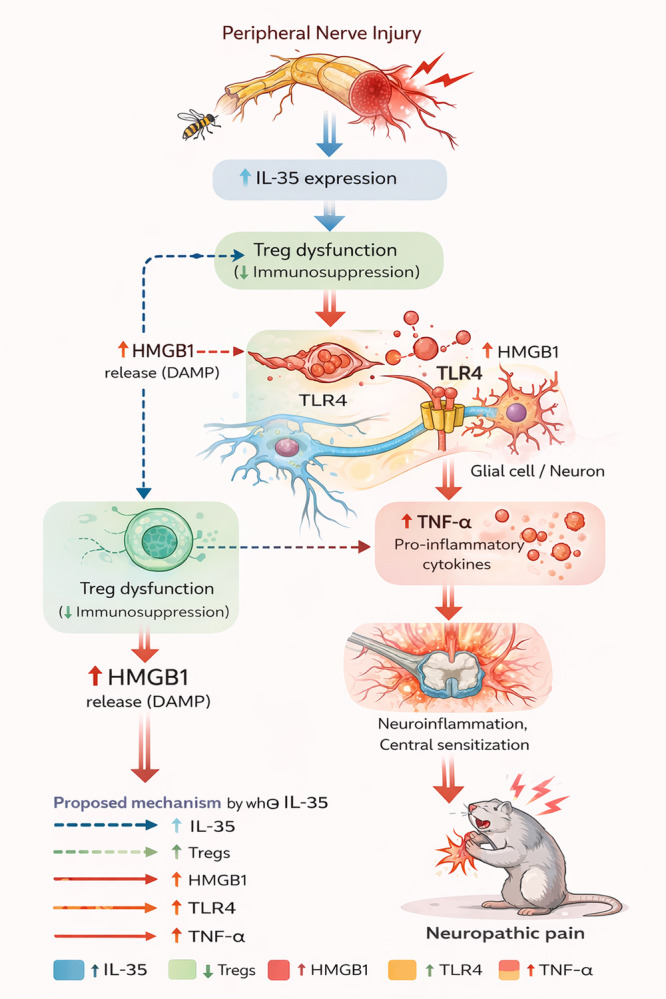
A schematic diagram illustrating the proposed mechanism by which reduced IL‐35 expression leads to regulatory T cell dysfunction and activation of the HMGB1/TLR4/TNF‐α inflammatory pathway in neuropathic pain.

A schematic diagram illustrating the proposed mechanism by which reduced IL‐35 expression leads to regulatory T cell dysfunction and activation of the HMGB1/TLR4/TNF‐α inflammatory pathway in neuropathic pain has been added as Figure 8.

## 4 Discussion

The treatment of neuropathic pain is a worldwide problem, and its pathogenesis and treatment have become one of the most challenging issues in medicine and biology [10]. At present, topical drugs such as antiepileptic drugs, antidepressants, opioids, and non‐steroidal anti‐inflammatory agents are often used in clinical practice to treat NP. Although they have certain clinical effects, more than half of the patients still cannot achieve satisfactory treatment [11]. In this study, a rat model of chronic compression injury of the sciatic nerve was prepared to simulate clinical neuropathic pain. By measuring MWT and TWL, it was found that the MWT and TWL of the NP group decreased after modeling, indicating that the rats had a pain response, proving that the model was successfully prepared. Therefore, this study uses neuropathic pain model rats to explore its pathogenesis, thereby providing a basis for exploring effective therapeutic drugs.

Recent advancements and deeper investigations into the mechanisms of neuropathic pain have revealed the pivotal role of immune cells and inflammatory cytokines in the onset and perpetuation of neuropathic pain [12]. T lymphocytes can mediate immune responses, and CD4+ and CD8+ cells in T lymphocyte subsets play an important role in stabilizing and regulating immune function. Ino et al. [13] found that in a rat model of neuropathic pain, there was a significant increase in T lymphocytes and natural killer cells surrounding the damaged nerve site. Malcangio et al. [14] further studied and found that compared with control wild‐type rats, the mechanical pain threshold and thermal pain hypersensitivity of sciatic nerve‐injured nude mice with missing mature T lymphocytes were significantly lower; while the T lymphocytes of wild‐type rats were extracted and injected into nude mice with T lymphocyte deficiency, it was found that the hyposensitivity to pain after nerve injury disappeared in the nude mice, which suggests that the inflammatory response after nerve injury may become a specific immune response mediated by lymphocytes. The findings of this study indicated that the CD4+ level in the whole blood of rats from the NP group was inferior compared to the Control group, while the CD8+ level was superior. Neuropathic pain is increasingly recognized as a neuroimmune disorder in which T lymphocytes play a critical modulatory role. Regulatory T cells (Tregs), through the secretion of immunosuppressive cytokines such as IL‐35, maintain immune homeostasis by suppressing excessive inflammatory responses. A reduction in Treg number or function leads to immune imbalance, sustained neuroinflammation, and enhanced pain sensitization.

IL‐35, an inherently immunosuppressive cytokine expressed by Tregs, can manifest its effects by attenuating inflammatory responses and modulating immune function [15]. Jiang et al. [16] discovered that IL‐35 mitigates diabetic neuropathic pain through the downregulation of the JNK signaling pathway. TNF‐α plays a crucial role as an inflammatory cytokine within the neuro‐endocrine‐immune functional system. Following nerve injury, activated immune cells and glial cells can generate substantial quantities of inflammatory factors, including TNF‐α [17]. Inflammatory cytokines increase neuronal excitation by binding to activated receptors on glial cells and neurons, thereby lowering pain thresholds by producing central sensitization [18]. Studies have found [19] that TNF‐α can inhibit the production and release of IL‐35, and it is speculated that TNF‐α may have a negative regulatory effect on the immunomodulatory effect of IL‐35. Toll‐like receptor 4 (TLR4) is a key receptor that recognizes and triggers corresponding central immune signals to affect the homeostasis of central nervous system immune function. Research has found [20] that the inflammatory response mediated by TLR4 and downstream cytokines plays an important role in the signal transduction process in NP rats. High‐mobility group protein 1 (HMGB1), as a nuclear factor, is released in inflammatory responses and can trigger inflammatory responses by interacting with TLR4. Previous studies [21] have established a mutual activation relationship between TLR4 and HMGB1. Extracellular HMGB1 interacts with TLR4 expressed on microglia and neurons, leading to activation of downstream NF‐κB signaling pathways. This interaction promotes the release of pro‐inflammatory cytokines such as TNF‐α, thereby amplifying neuroinflammation and exacerbating neuropathic pain [21]. This study revealed a significant decrease in the serum level of IL‐35, as well as reduced expression of IL‐35 protein and mRNA in the spinal cord tissue of rats in the NP group. Conversely, the expression of TNF‐α, TLR4, and HMGB1 protein and mRNA in spinal cord tissue exhibited a significant increase, suggesting that IL‐35 may mediate the occurrence of neuropathic pain by regulating T cell function and inflammatory response.

## 5 Strengths and Limitations

This study provides novel insights into the role of IL‐35 in neuropathic pain by demonstrating, through a combination of behavioral, histological, and molecular techniques, that decreased IL‐35 expression is associated with enhanced inflammatory responses and immune dysregulation in a rat model of chronic constriction injury. A key strength of this work lies in its comprehensive and methodologically diverse approach, which integrates systemic immune profiling with localized spinal cord analysis, thereby strengthening the validity and relevance of the findings. However, the study also has limitations. As it is based solely on animal experiments, the results may not fully translate to human neuropathic pain. Furthermore, the research did not employ functional interventions, such as IL‐35 overexpression or blockade, to directly confirm causality. Mechanistic pathways through which IL‐35 influences specific cellular populations like glial cells were not explored, and the time frame was limited to a single post‐injury stage, potentially overlooking dynamic changes over time.

## 6 Conclusion

This study demonstrates that IL‐35 expression is significantly downregulated in a rat model of neuropathic pain induced by chronic constriction injury, and this reduction correlates with an increase in pro‐inflammatory markers, including TNF‐α, TLR4, and HMGB1, within the spinal cord. Concurrently, alterations in T cell subsets were observed, characterized by a reduction in CD4⁺ T cells and an elevation in CD8⁺ T cells, indicating a shift toward an immune profile that favors inflammation and contributes to the maintenance of neuropathic pain. These findings suggest that IL‐35 plays a crucial regulatory role in modulating both systemic and local immune responses during the development of neuropathic pain.

## Author Contributions

Conception and design: Pingdi You and Sunzhi Lin. Method: Lirui Shen, Zhixiong Huang, and Cuihua Yuan. Data collection: Cuihua Wei, Bili Wang, and Yongwu Chen. Manuscript writing: Pingdi You. Manuscript revision: Sunzhi Lin. Research supervision: Sunzhi Lin. All authors contributed to the article and approved the submitted version.

## Ethics Statement

All animal procedures were carried out in compliance with the guidelines for scientific animal procedures approved by the ethics committee of the Mindong Hospital Affiliated to Fujian Medical University, and the approval number is No. 2021032536H.

## Consent

The authors have nothing to report.

## Conflicts of Interest

The authors declare no conflicts of interest.

## Data Availability Statement

The data that support the findings of this study are available from the corresponding author upon reasonable request.
